# Evaluation of Wall Correction Factor of INER's Air-Kerma Primary Standard Chamber and Dose Variation by Source Displacement for HDR ^192^Ir Brachytherapy

**DOI:** 10.1155/2013/436979

**Published:** 2013-10-08

**Authors:** J. H. Lee, J. N. Wang, T. T. Huang, S. H. Su, B. J. Chang, C. H. Su, S. M. Hsu

**Affiliations:** ^1^Health Physics Division, Institute of Nuclear Energy Research, Longtan 325, Taiwan; ^2^Department of Radiation Oncology, China Medical University Hospital, Taichung 404, Taiwan; ^3^Department of Biomedical Imaging and Radiological Sciences, National Yang-Ming University, Taipei 112, Taiwan

## Abstract

The aim of the present study was to estimate the wall effect of the self-made spherical graphite-walled cavity chamber with the Monte Carlo method for establishing the air-kerma primary standard of high-dose-rate (HDR) ^192^Ir brachytherapy sources at the Institute of Nuclear Energy Research (INER, Taiwan). The Monte Carlo method established in this paper was also employed to respectively simulate wall correction factors of the ^192^Ir air-kerma standard chambers used at the National Institute of Standards and Technology (NIST, USA) and the National Physical Laboratory (NPL, UK) for comparisons and verification. The chamber wall correction calculation results will be incorporated into INER's HDR ^192^Ir primary standard in the future. For the brachytherapy treatment in the esophagus or in the bronchi, the position of the isotope may have displacement in the cavity. Thus the delivered dose would differ from the prescribed dose in the treatment plan. We also tried assessing dose distribution due to the position displacement of HDR ^192^Ir brachytherapy source in a phantom with a central cavity by the Monte Carlo method. The calculated results could offer a clinical reference for the brachytherapy within the human organs with cavity.

## 1. Introduction

In radiotherapy treatments for cancer patients it is critical to have an accurate measurement of the dose delivered to the patient. Obtaining an accurate dose involves three major steps. The first step is the establishment of primary standards of air kerma or absorbed dose to water. The second step is the use of dosimetry protocols based on ion chambers calibrated using these primary standards to establish the dose under reference conditions in a clinical therapy beam. The final step is to establish the dose distribution in individual patients specified by computed tomography (CT) data.

At present, ^192^Ir is the most commonly used radioisotope for high-dose-rate (HDR) brachytherapy treatment. Due to the relatively short half-life of ^192^Ir (73.827 days ± 0.013 days) [1], most radiotherapy departments change their ^192^Ir sources every three months and medical physicists need to measure the source strength of the ^192^Ir sources on a regular basis before an accurate treatment plan can be written. The source calibration is a main component of quality assurance programs recommended for HDR brachytherapy [2]. The recommended quantity for the specification of brachytherapy gamma ray sources is the reference air-kerma rate (RAKR), defined by the International Commission on Radiation Units and Measurements [3, 4] as the kerma rate to air, in air, at a reference distance of 1 meter, corrected for air attenuation and scattering.

To replace the interpolation techniques between air-kerma calibration coefficients of an ionization chamber for ^192^Ir [5, 6], the Institute of Nuclear Energy Research (INER, Taiwan) has recently developed a spherical graphite-walled cavity ionization chamber as the primary standard for direct measurement of HDR ^192^Ir brachytherapy source strength to provide a traceable source calibration. Our primary standard is derived from measurements using the graphite-walled chamber, based on the Bragg-Gray theory. The wall correction factor *k*_wall_ is intended to account for the effects of attenuation and scatter of the incident primary photons in the chamber wall [7]. The *k*_wall_ of the primary standard chamber for HDR ^192^Ir brachytherapy sources is the largest correction factor (approximately 80% of the total correction amounts) required to correct the measured charge for experimental perturbations. In the past, the empirical method to estimate *k*_wall_ has been to measure the ionization charge (or current) as a function of wall thickness for a fixed cavity size (but for wall thickness no smaller than the minimum required to exclude secondary electrons generated outside the wall). The results are then linearly extrapolated to zero wall thickness under the assumption that attenuation and scattering are thus eliminated. For more than a decade, Rogers and Bielajew suggested that the use of *k*_wall_ based on linear extrapolation measurements was incorrect and proposed instead the use of results from the Monte Carlo calculations [8–12].

In this research, one of the tasks was to evaluate *k*_wall_ of the self-fabricated chamber by the Monte Carlo photon-electron transport calculations to establish the HDR ^192^Ir air-kerma primary standard at INER. In clinical brachytherapy treatment of the esophagus or bronchi or such organs with a cavity, the source positions tend to displace because there would be no body tissue to fix the isotope. The fact is that the position of the isotope would affect the accuracy of the output dose. So the source displacement would cause the tumor or normal tissue to receive inaccurate dose and deviate from the treatment plan and desired effect. Since the experiment of actually measuring the dose variations in the human body is not feasible, we used a simulation calculation to perform dose evaluation [13–15]. In this research, we also performed sensitivity assessment using the Monte Carlo method for the displacement of an HDR ^192^Ir source in the cavity and explored how the related effects would affect internal doses. Hopefully the evaluation results will offer a clinical reference for brachytherapy within human organs with a cavity.

## 2. Materials and Methods

### 2.1. Wall Correction Factor Calculation for HDR ^192^Ir Air-Kerma Standard Chamber

**Figure 1:**
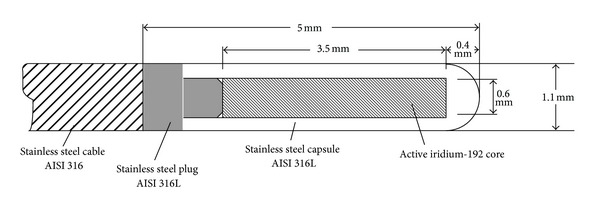
Geometric model of the Nucletron mircoSelectron HDR ^192^Ir Classic source.

The INER uses a Nucletron microSelectron HDR Classic brachytherapy unit fitted with the “Classic” source, part number 096.001, manufactured by Mallinckrodt Medical B V (The Netherlands). The average photon energy for HDR ^192^Ir brachytherapy sources is close to 0.4 MeV [16].  Figure 1 shows a schematic diagram of the HDR ^192^Ir brachytherapy source simulated in this work. The enclosure of the radioactive material consists of a cylindrical stainless steel AISI 316L capsule (length: 5.0 mm, radial thickness: 250 *μ*m) which is sealed by laser welding. The ^192^Ir is contained in the capsule as a metallic ^192^Ir cylinder (length: 3.5 mm, diameter: 0.6 mm). The stainless steel capsule is welded to a metal plug and a 1500 mm long flexible stainless steel AISI 316 cable. The other end of the capsule is welded to a steel pin (tail). The identification of the source is engraved on the long side of the tail. The nominal initial activity of the source is between 370 GBq and 550 GBq. 

**Figure 2:**
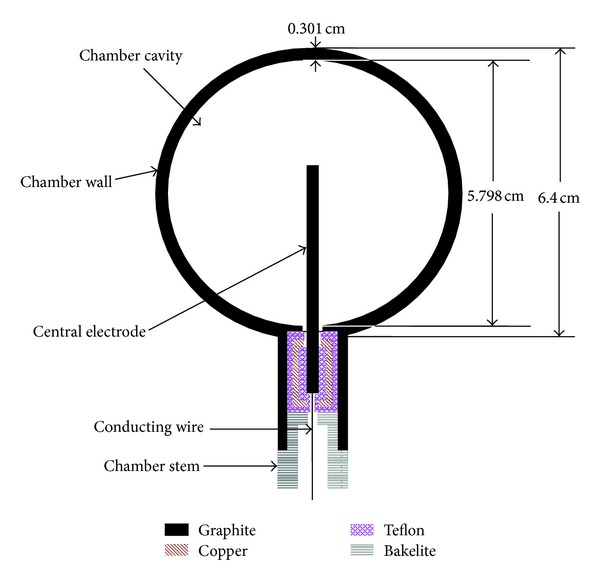
Schematic of INER primary standard cavity chamber for HDR ^192^Ir source.

**Figure 3:**
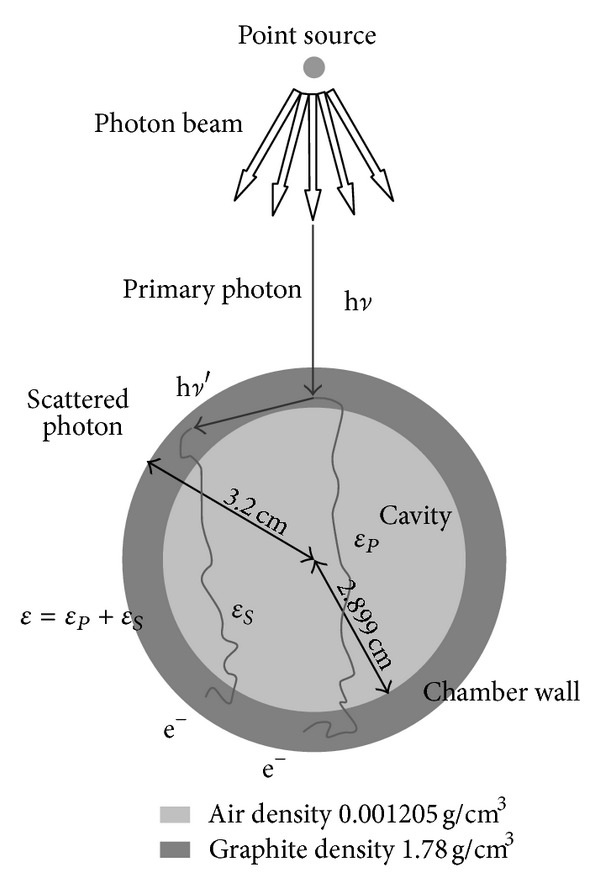
The Monte Carlo simulation geometry for wall correction assessment of the INER primary standard cavity chamber for HDR ^192^Ir source.

The INER primary standard cavity chamber for HDR ^192^Ir sources, shown in Figure 2, is a guarded ionization chamber resulting in low leakage currents. The spherical cavity volume of the primary standard chamber was measured on two coordinate measuring machines and found to be 102 cm^3^. The outside radius (3.200 cm), inside radius (2.899 cm), and wall thickness (0.301 cm) for the chamber were measured with a similar technique. In the case of ^192^Ir, this requires a wall thick enough to stop 687 keV Compton recoil electrons generated by 885 keV gamma rays, the most energetic photons emitted by ^192^Ir [17], neglecting three very weak lines above 1 MeV. The CSDA (continuous slowing down approximation) range of 687 keV electrons is 0.31 g cm^−2^ of graphite [18], which is equivalent to a wall thickness of approximately 1.8 mm. The graphite wall of this cavity chamber provides sufficient build-up material to ensure CPE. The computer code CAVSPHnrc, which is a user code of EGSnrc [19], is used to calculate wall correction factors of standard cavity chambers. The ionization chamber used in the calculation comprised two concentric spheres, and if ignoring the central electrode, there would be three areas including air, graphite wall, and cavity (made of air) from the outside to the inside. It was assumed that the ^192^Ir source was a point source and that the distance between the source and ionization chamber was 100 cm. The simulation kept track of the wall from which the electrons entered the cavity and calculated *k*_att_ and *k*_sc_, the correction factors for attenuation and scattering in the wall. From Figure 3 the following equations become evident:

(1) $$\begin{array}{c} k_{\text{wall}}=k_{\text{att}}\cdot k_{\text{sc}}=\frac{\langle e^{+\mu \text{t}}\varepsilon_{P}\rangle }{\langle \varepsilon_{P}+\varepsilon_{S}\rangle }=\frac{\langle e^{+\mu \text{t}}\varepsilon_{P}\rangle }{\langle \varepsilon \rangle }, \end{array}$$

(2) $$\begin{array}{c} k_{\text{att}}=\frac{\langle e^{+\mu \text{t}}\varepsilon_{P}\rangle }{\langle \varepsilon_{P}\rangle }, \end{array}$$

(3) $$\begin{array}{c} k_{\text{sc}}=\frac{\langle \varepsilon_{P}\rangle }{\langle \varepsilon_{P}+\varepsilon_{S}\rangle }=\frac{\langle \varepsilon_{P}\rangle }{\langle \varepsilon \rangle }, \end{array}$$

where the primary photon collides with the chamber wall and produces electrons and a scattered photon, 〈*ε*_*P*_〉 is the energy deposition caused by the primary photon, 〈*ε*_*S*_〉 is the energy deposition caused by the scattered photon, and 〈*ε*〉 = 〈*ε*_*P*_ + *ε*_*S*_〉 which stands for all the energy deposition inside the chamber cavity. If the ionization chamber was wall-less, the energy deposition in the cavity would be the energy deposition of the primary photon before attenuation, that is, 〈*e* ^+*μt*^ *ε*_*P*_〉. Hence, the definition of the wall correction factor, *k*_wall_, would be the ratio of the actual energy deposition 〈*e* ^+*μt*^ *ε*_*P*_〉 to the wall-less energy deposition 〈*ε*〉. In order to produce results that could be used for arbitrary beam spectra, calculations for the self-made chamber were done for both monoenergetic and spectral photon beams. Samples of at least 10^8^ incident primary photons were used to make the relative statistical standard deviations below 0.1%.

### 2.2. Assessment of Dose Variation by Source Displacement for HDR ^192^Ir Brachytherapy

**Figure 4:**
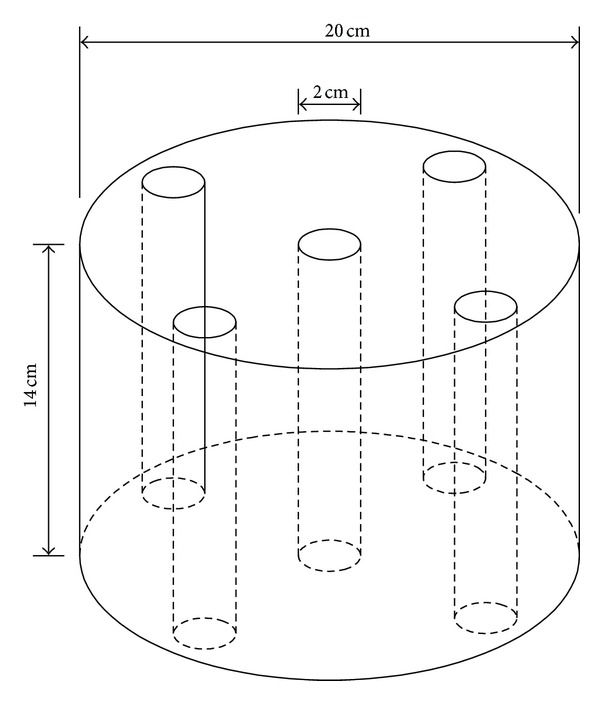
Diagram of Plexiglass phantom geometry.

**Figure 5:**
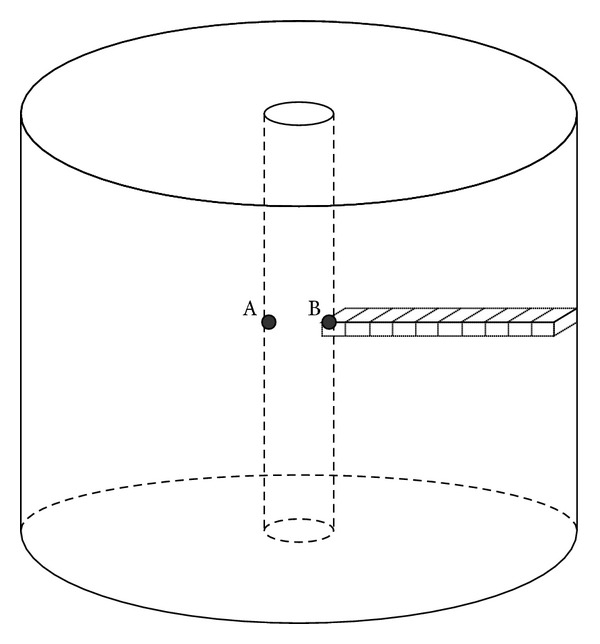
Geometrical indication for the positions of HDR ^192^Ir source and dose assessment scoring volume in Plexiglass phantom.

Plexiglass is a useful phantom material for experimental measurement and calibration and can be easily custom-made for certain shapes and purposes. The radiation interaction properties and density of Plexiglass are similar to the human soft tissue. The Plexiglass phantom is a cylinder with a height of 14 cm and a radius of 10 cm, as indicated in Figure 4. In the center of the phantom, there is a 2 cm diameter cylindrical cavity, which in this calculation represents human organs with a cavity such as the esophagus and bronchi. At the edge of the Plexiglass phantom there are also four cylindrical cavities of the same size as that of the central one. In this calculation the cavities at the edge were not simulated and were filled with the phantom material. To simulate the movement of the ^192^Ir HDR source in the cavity, the source was located at the cylindrical cavity surface in the calculation, at point A and point B in Figure 5. The HDR ^192^Ir source was placed at a height of 7 cm in the cavity, which is the middle of the cavity length. As Figure 5 showed, the distance between point A and B is exactly the diameter of the cylindrical cavity. The photon and electron radiations were simulated separately with a Nucletron microSelectron Classic source with an activity of 3.552 × 10^11^Bq (9.6 Ci) and then the doses due to photon and electron radiations were summed. The geometrical model of the dose monitoring positions is also indicated in Figure 5, taking the cube whose side length was 0.5 cm as a scoring volume. The monitoring positions went along as a straight line from the radius of the central cavity to the side surface of Plexiglass phantom. In this work, the doses at the same monitoring points while the ^192^Ir source was positioned at the central axis of the cavity were also calculated as the comparison basis for the impact of the source displacement.

## 3. Results and Discussions

### 3.1. Calculation and Verification of the Chamber Wall Correction Factors

**Figure 6:**
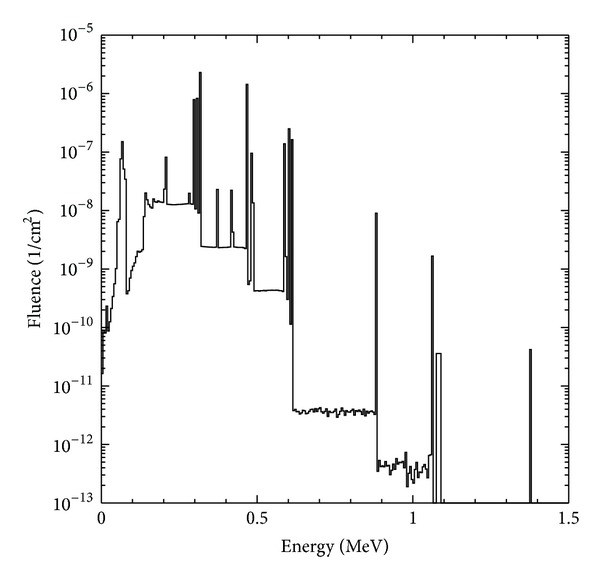
Fluence spectrum for the Nucletron mircoSelectron HDR ^192^Ir Classic source at the RAKR measurement point.

RAKR is determined in air at a distance of 1 m from the source axis in the plane that perpendicularly bisects the axis. The photon spectrum at the RAKR measurement point was estimated by assuming the photon emission probabilities for ^192^Ir decay and calculating the attenuated spectrum reaching the measurement point. The calculation took into account the attenuation along all photon paths through the various materials by integrating overall source points in the cylindrical core. The photon intensity and spectrum for the source at the RAKR determination point was calculated using MCNP code version 5 [20]. In the calculation the geometry model of the HDR ^192^Ir source was constructed and simulated in detail. The energy cut-off settings for the photon and electron transport were both 0.001 MeV. Photons were sampled uniformly in the core and were scored at the side of the HDR ^192^Ir source.  Figure 6 shows the ^192^Ir source photon spectrum calculated in this research, with which the spectrum for the similar field from the independent Monte Carlo calculations of Rogers and Borg [21] showed a good agreement.

**Figure 7:**
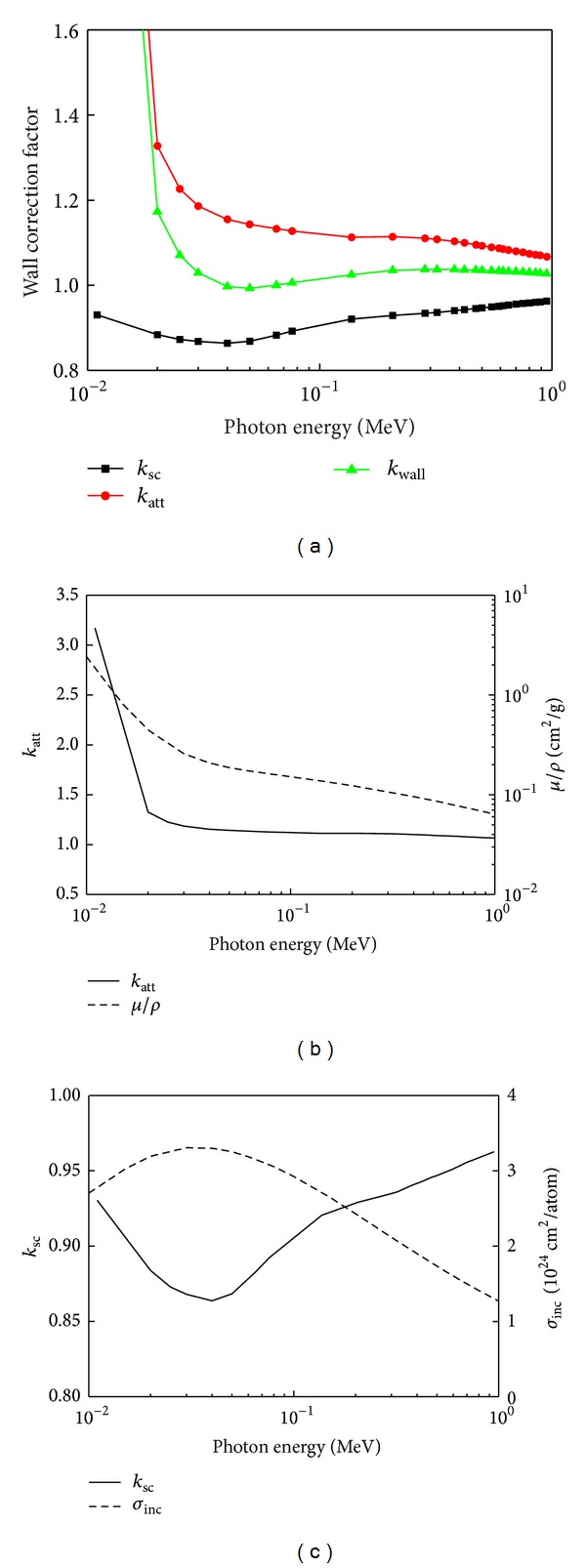
(a) Wall correction factors *k*_wall_, attenuation correction factor *k*_att_, and scatter correction factor *k*_sc_, (b) *k*_att_ and mass attenuation coefficient of graphite *μ*/*ρ*, and (c) *k*_sc_ and incoherent scattering cross section *σ*_inc_ as functions of the incident photon energy.

The ^192^Ir source photon spectrum evaluated in Figure 6 was taken as the input data of the CAVSPHnrc code and was used to calculate the wall correction factors of INER's spherical graphite-wall cavity chamber. The *k*_wall_, *k*_att_, and *k*_sc_ calculation values and simulation uncertainty analysis for INER's HDR ^192^Ir primary standard chamber are listed in Table 1.  Figure 7(a) illustrates the wall correction factors and the related parameters of INER's spherical graphite-wall cavity chamber, *k*_wall_, *k*_att_, and *k*_sc_, as functions of the incident photon energy. The *k*_att_ value reduced with the increase of energy. When compared with the mass attenuation coefficient (*μ*/*ρ*) of graphite, it showed similar variation trends (Figure 7(b)) and met the proportional relationship between *k*_att_ and *e* ^+*μt*^ in (2). *k*_sc_ varied inversely with the incoherent scattering cross section (Figure 7(c)) related to the energy deposition caused by the scattered photon 〈*ε*_*s*_〉 in (3). The smallest *k*_sc_ value occurred at 0.04 MeV. The wall correction factors *k*_wall_ affected by the related parameters have been established in this study and will be incorporated into INER's air-kerma primary standard for HDR ^192^Ir sources.

INER used the CAVSPHnrc code to calculate the *k*_wall_ values of NIST's spherical graphite primary standard chambers of different volumes for 0.40 MeV, 0.662 MeV, and 1.25 MeV photons and compared them with the results evaluated by NIST [22]. The comparison results are listed in Table 2. Table 2 shows that the *k*_wall_ assessment discrepancies against the NIST primary standard chambers between INER and NIST for monoenergetic photons were within 0.20%, verifying the accuracy and reliability of INER's method for evaluating the chamber wall correction factors. The Monte Carlo simulation process in this study also offered a convenient and accurate approach to evaluate the chamber wall correction factor for national metrology institutes (NMIs) in establishing their air-kerma primary standards of gamma radiation.  Table 3 gives the structural information of ^192^Ir primary standard cavity chambers for INER, NIST (50cc-1), and NPL [1, 22]. INER used the Nucletron microSelectron Classic source spectrum simulated in Figure 6 and the CAVSPHnrc code to calculate NIST's and NPL's ^192^Ir primary standard chamber wall correction factors given in Table 4. It can be seen from Table 4 that for the assessment of wall correction factors, the difference between evaluations of NPL's standard chamber was 0.23% and between evaluations of NIST's standard chamber was 0.44%. The differences were analyzed and the root cause was found: the HDR ^192^Ir source used by the NPL was the Nucletron microSelectron Classic type [16], which was the same type used by INER for calculation of the HDR ^192^Ir source spectrum. On the other hand, NIST used a low-dose-rate (LDR) ^192^Ir brachytherapy seed source to evaluate the wall correction factor [22]. With the different types of sources, INER and NIST had a larger difference in the evaluation results. Analyzing Tables 2 and 4, it can be seen that for the NIST 50cc-1 primary standard chamber for an ^192^Ir source, the *k*_wall_ evaluation difference of the ^192^Ir spectral photon beams is higher than that for the monoenergetic photons (0.40 MeV, 0.662 MeV, and 1.25 MeV).

### 3.2. Assessment of Dose Variation by Source Displacement

**Figure 8:**
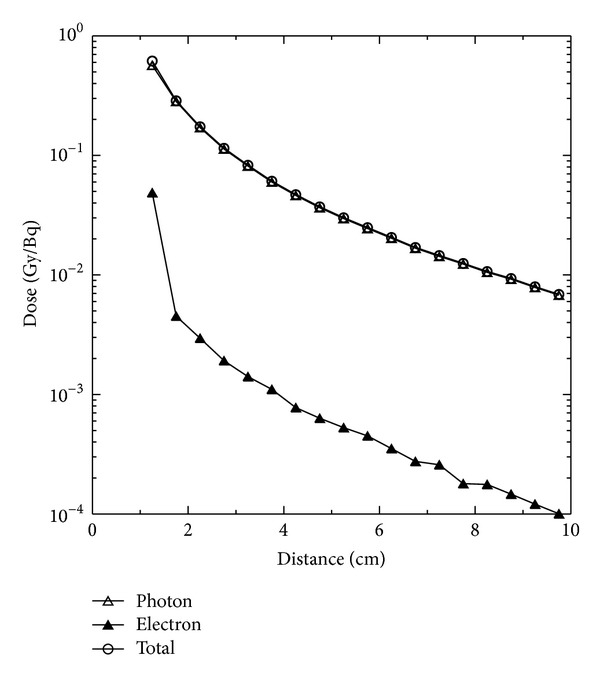
Indication of the dose distribution varying with assessment distance while HDR ^192^Ir source was placed at the central axis of the Plexiglass phantom cylindrical cavity.

The MCNP code version 5 was adopted to evaluate the influence on the dose distribution of the phantom for HDR ^192^Ir brachytherapy source movement in the central cavity of the Plexiglass phantom.  Figure 8 indicates the photon dose, electron dose, and the total dose as a function of distance when the source was placed on the central axis of the central cylindrical cavity. It is obvious that the dominant dose contribution came from the photons of the source. At the distance of 1.25 cm from the central cavity axis, the electron dose from the source was about 1/7 of the photon dose. At other distance monitoring points, the dose contribution of the electrons was only about 1/60 of the photon dose. The difference of these compared results as a function of distance was caused by the short range of the electrons. The electrons deposit energy quickly as they transport through 0.5 cm depth of the phantom, and the electron dose at the phantom edge results from the Bremsstrahlung. The dose contribution from the Bremsstrahlung was estimated to be approximately 17% of the total electron dose summed from all monitoring points. The dose assessment in the Plexiglass phantom showed that the total dose on the surface of the phantom was as low as only 1% of that at the phantom center.

**Figure 9:**
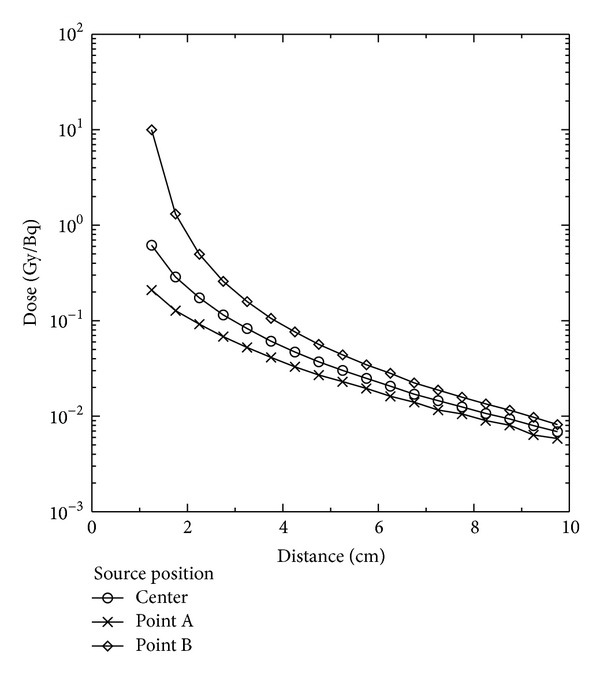
Indication of the total dose distribution varying with assessment distance while the HDR ^192^Ir source was placed at different locations in the Plexiglass phantom cylindrical cavity.

Figure 9 indicates that the total dose distribution varied with assessment distance when the ^192^Ir source was placed at different locations in the cylindrical cavity of the Plexiglass phantom. The dominant dose contribution is still from the photons, even if the location of the ^192^Ir source has been displaced.  Table 5 lists the dose ratios at various monitoring points when the ^192^Ir source was placed at the point A, point B, and the central axis of the phantom cavity center. It was seen that for the nearest monitoring point (1.25 cm), the dose ratios of which the source was located at point A and point B against the source located at the cavity central axis were 0.340 and 16.2, respectively. The analysis results showed that when the location of the ^192^Ir source was displaced from point A to point B, the dose at the nearest motoring point (1.25 cm) was greater by nearly a factor of 50. The dose evaluation difference from the location of the ^192^Ir source decreased as the monitoring distance increased. At the edge of the Plexiglass phantom (9.75 cm), the dose difference was reduced to 28.5%. From the above calculation results, it can be known that when performing brachytherapy for organs with a cavity, the displacement of source position could make the dose output very different from the plan. With the increase of the assessment distance, the impact from the source displacement would be greatly reduced. However, for clinical brachytherapy, the source is usually very close to the tumor. Source position displacement in organs with cavities such as the esophagus and the bronchi may cause the delivered dose to differ from the prescribed dose in the treatment plan and may bring unexpected influence to the treatment results.

## 4. Conclusions

We calculated *k*_wall_ for a self-made spherical chamber using the Monte Carlo method. The wall correction factor could be applied in the establishment of an air-kerma primary standard for HDR ^192^Ir brachytherapy sources. Simulation comparisons for the primary standard chamber wall corrections of different laboratories were employed to verify the accuracy of the assessment approach established by INER. The comparison results showed that INER and NPL had a better agreement with wall correction factor evaluation of NPL's standard chamber using the same type of source as compared with the different type of ^192^Ir source used with NIST's standard chamber, which resulted in an increased discrepancy. The *k*_wall_ calculation data in this study will be incorporated into INER's air-kerma primary standard for HDR ^192^Ir sources in the near future and increase the calibration accuracy in brachytherapy source strength measurements.

According to the comparison results of the dose distribution of the Plexiglass phantom for the HDR ^192^Ir brachytherapy source, the outcome of brachytherapy will be seriously influenced by the source displacement. When dealing with clinical treatments including an internal cavity such as esophagus or bronchi, doctors and medical physicists should pay special attention to the dose output variations caused by the source that could not be exactly fixed. The MCNP simulation model used in this research hopefully could be applied to the Voxel phantom which is constructed by CT images to offer more contributions to improve clinical patient treatments.

## Figures and Tables

**Table 1:** The *k*_wall_, *k*_att_, and *k*_sc_ calculation values and simulation uncertainty analysis for INER's HDR ^192^Ir primary standard chamber.

Correction factors	*k*_ sc_	*k*_ att_	*k*_ wall_
Calculation values	0.9402	1.1021	1.0362
Simulation uncertainties	0.015%	0.005%	0.015%

**Table 2:** Comparison of *k*_wall_ values calculated by INER and NIST for NIST's spherical graphite primary standard chambers using 0.40 MeV, 0.662 MeV, and 1.25 MeV photons.

Chamber	*k*_wall_ (0.40 MeV)			*k*_wall_ (0.662 MeV)			*k*_wall_ (1.25 MeV)		
	NIST	INER	Difference	NIST	INER	Difference	NIST	INER	Difference
1cc	1.0312	1.0320	0.07%	1.0286	1.0287	0.01%	1.0197	1.0287	0.01%
10cc	1.0349	1.0352	0.03%	1.0314	1.0319	0.05%	1.0226	1.0319	0.05%
30cc	1.0374	1.0395	0.20%	1.0348	1.0344	−0.04%	1.0249	1.0344	−0.04%
50cc-1	1.0386	1.0402	0.16%	1.0349	1.0354	0.05%	1.0252	1.0354	0.05%

**Table 3:** Structural characteristics of ^192^Ir primary standard cavity chambers for INER, NIST (50cc-1), and NPL.

Laboratory	Graphite density (g cm^−3^)	Inside radius (cm)	Outside radius (cm)	Wall thickness (cm)	Cavity volume (cm^3^)
INER	1.78	2.899	3.200	0.301	102
NIST	1.73	2.305	2.670	0.365	51.3
NPL	1.75	2.910	3.290	0.380	103

**Table 4:** Calculation comparison for wall correction factors of the ^192^Ir primary standard chambers for NPL and NIST.

The NPL primary standard chamber for ^192^Ir			
Wall correction factors calculated by	NPL	INER	Difference
*k*_ wall_ values	1.0453	1.0429	−0.23%

The NIST primary standard chamber (50cc-1) for ^192^Ir			
Wall correction factors calculated by	NIST	INER	Difference
*k*_ wall_ values	1.0349	1.0395	0.44%

**Table 5:** Total dose distribution assessments when ^192^Ir source was at different locations.

Distance between central axis and monitoring point (cm)	Dose ratios of monitoring points when source was at different locations		
	Point A/central axis	Point B/central axis	Point A/point B
1.25	0.340	16.2	0.021
1.75	0.445	4.57	0.097
2.25	0.530	2.86	0.185
2.75	0.592	2.25	0.263
3.25	0.633	1.91	0.331
3.75	0.676	1.74	0.390
4.25	0.700	1.62	0.431
4.75	0.726	1.52	0.478
5.25	0.764	1.46	0.525
5.75	0.784	1.39	0.565
6.25	0.783	1.36	0.574
6.75	0.825	1.31	0.628
7.25	0.797	1.29	0.618
7.75	0.844	1.27	0.666
8.25	0.842	1.26	0.668
8.75	0.860	1.24	0.696
9.25	0.797	1.22	0.652
9.75	0.848	1.19	0.715
